# Choosing Between Short-Read 16S, Full-Length ONT 16S, and Long-Read Shotgun Metagenomics for Soil Microbiome Studies: A Critical Review of the Benchmarking Evidence

**DOI:** 10.3390/microorganisms14051132

**Published:** 2026-05-16

**Authors:** Abdulla Albastaki, Judith Smith

**Affiliations:** 1 General Department of Forensic Science and Criminology, Dubai Police HQ, Dubai P.O. Box 24954, United Arab Emirates; 2 School of Law and Policing, University of Lancashire, Preston PR1 2HE, UK

**Keywords:** soil microbiome, 16S rRNA, Oxford Nanopore Technologies, shotgun metagenomics, Illumina, sequencing bias, benchmarking

## Abstract

Studying soil microbiomes is challenging because soil contains thousands of microbial species at vastly different abundances. The choice of sequencing method has a strong effect on which of these species are detected and how the community is described. Three approaches now dominate soil microbiome research: short-read 16S rRNA amplicon sequencing on Illumina platforms, full-length 16S sequencing on Oxford Nanopore Technologies (ONT) platforms (particularly the R10.4.1 flow cell), and long-read shotgun metagenomics. Each has distinct biases that shape the recovered community, yet researchers routinely select a method based on cost, understanding, or local expertise rather than on a clear knowledge of what each approach methodically over- or under-represents. Here, we review head-to-head benchmarking studies that have applied two or more of these methods to the same soil or directly comparable samples. We show that while long-read and short-read 16S approaches generally converge on dominant taxa and on between-sample differences, they disagree substantially on alpha diversity estimates, rare taxon detection, and the relative abundances of entire phyla. The R10.4.1 flow cell chemistry has narrowed but not eliminated the accuracy gap with Illumina, and shotgun metagenomics reveals systematic biases in both short and long-read assembly that depend on population diversity within the sample. We synthesise this evidence into an evidence-based decision framework tied to specific research questions and recognise the gaps in soil-specific benchmarking that limit current methods. Rather than asking which platform is “best,” we argue that method choice should be framed as an important part of study design, with the biases of the chosen method acknowledged and, where possible, controlled for.

## 1. Introduction

Soil microbial communities mediate processes that are essential for ecosystem function, including nutrient cycling, organic matter decomposition, plant health, and global biogeochemistry. A single gram of soil can contain on the order of 10^9^ to 10^10^ microbial cells and thousands of different taxa, most of which cannot be cultured under standard laboratory conditions [1,2]. This combination of high biomass, extreme diversity, and low-abundance organisms makes soil the most analytically challenging of all microbial habitats, and it is in soil that differences between sequencing platforms most clearly explain variances in the biological conclusions that scientists reach.

**Figure 1:**
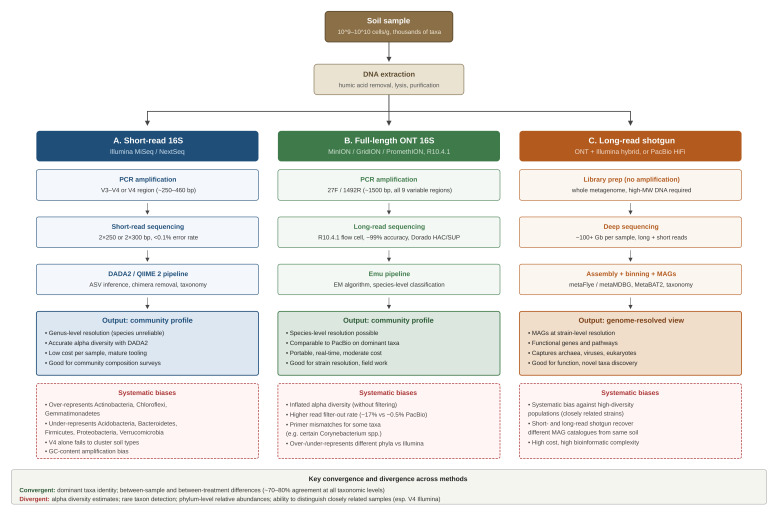
Conceptual workflow comparison of the three sequencing strategies reviewed. Starting from a single soil sample and a shared DNA extraction step, the workflow diverges into (**A**) short-read 16S amplicon sequencing on Illumina platforms, targeting the V3–V4 or V4 hypervariable region; (**B**) full-length 16S rRNA gene sequencing on Oxford Nanopore Technologies platforms with R10.4.1 flow cell chemistry, targeting all nine variable regions; and (**C**) long-read or hybrid long-plus-short-read shotgun metagenomic sequencing of the entire metagenome. For each workflow, the figure summarises the principal output (community profile or genome-resolved view) and the systematic biases that affect interpretation, drawn from the head-to-head benchmarking evidence reviewed.

High-throughput sequencing has, over the past two decades, replaced cultivation as the most dominant way to look at soil microbiomes. Three strategies now structure the field, including short-read 16S rRNA amplicon sequencing, typically of the V3–V4 or V4 hypervariable regions on Illumina platforms, and remains the most widely used approach because of its low per-sample cost and high per-base accuracy; full-length 16S sequencing on third-generation platforms, primarily Oxford Nanopore Technologies (ONT) and Pacific Biosciences (PacBio) covers the entire 1500 bp 16S gene and, in principle, enables species- and strain-level resolutions that short-reads cannot achieve; and shotgun metagenomic sequencing, increasingly performed with long-reads, which sequences the entire DNA in a sample and allows functional profiling and recovery of metagenome-assembled genomes (MAGs). Figure 1 offers a schematic overview comparing the three sequencing approaches.

Many recent reviews have described these technologies and their bioinformatic pipelines in detail [3,4]. However, far less attention has been paid to a question that every soil microbiome researcher ultimately has to answer: when the same DNA extract is processed through different methods, do we get the same picture of the community? The answer turns out to be more complex than either the early literature on long-read sequencing or the conservative continued reliance on short-read 16S would suggest. In a recently published three-platform benchmark of three soil types, ONT R10.4.1 and PacBio produced broadly comparable bacterial diversity assessments, with PacBio showing slightly higher efficiency in detecting low-abundance taxa, while the V4 region on Illumina alone failed to cluster samples by soil type at all [5]. In an earlier two-platform benchmark on agricultural soils, the sequencing platform had a substantial influence on the relative abundances of taxa, with MiSeq resulting in significantly higher abundances of Actinobacteria, Chloroflexi, and Gemmatimonadetes, and lower abundances of Acidobacteria, Bacteroidetes, Firmicutes, Proteobacteria, and Verrucomicrobia compared to the MinION platform [6]. These are not minor differences, and are considered major phylum-level differences in what the soil looks like, which was clearly driven by the choice of instrument and primers.

The purpose of this review is therefore not to review sequencing technologies, which has been done repeatedly and recently [3,4], but to critically synthesise what the head-to-head benchmarking literature tell us about where soil microbiome results converge across methods and where they systematically diverge. We focus on three dominant strategies: short-read 16S on Illumina, full-length 16S on ONT (with particular emphasis on R10.4.1 chemistry), and shotgun metagenomics with long or hybrid long-plus-short-reads. We deliberately exclude PacBio-only methodology discussions from the central argument because PacBio is currently inaccessible to most soil microbiome laboratories due to cost, though PacBio data from benchmark studies serve as a useful reference point. We argue that the choice of sequencing method is not a neutral technical decision but a study design decision that shapes the biological conclusions a paper can support, and we propose an evidence-based framework to help researchers make this choice clearly rather than by default. This review aims to (i) synthesise the published head-to-head benchmarking evidence comparing short-read 16S, full-length ONT 16S, and long-read shotgun metagenomic sequencing on matched soil or directly comparable samples; (ii) critically assess the extent to which the R10.4.1 flow cell chemistry has narrowed the accuracy gap between ONT and Illumina; and (iii) propose an evidence-based framework for method selection that is tied explicitly to the research question being asked.

## 2. Scope, Evidence Base, and Review Methodology

This review synthesises benchmarking studies published between 2020 and 2025 in which two or more of the three sequencing strategies (short-read 16S, full-length ONT 16S, long-read shotgun) were applied to the same samples or to comparable soil samples. Where soil-specific benchmarks were not available for a particular comparison (for example, direct tests of the R10.4.1 chemistry improvement), we include benchmarking evidence from non-soil samples and note the limits of this generalisation.

The evidence base consists of four categories of study. First, direct three-platform benchmarks applied to soil samples, which provide the strongest evidence for method choice in soil work [5,6]. Second, two-platform benchmarks comparing long- and short-read 16S in other complex microbial samples, which notify the methodological picture but require care in estimation [7,8,9]. Third, studies focused specifically on the R10.4.1 chemistry and its predecessors, which look into the accuracy and error estimates of the current ONT platform [10,11]. Fourth, studies of long-read and hybrid shotgun metagenomics applied to soil, which address MAG recovery and functional profiling [12,13,14].

A recent review in *Frontiers in Soil Science* by Reznikova and colleagues provided an extensive methodological outline of next-generation sequencing for soil microbiomes, including a comprehensive survey of bioinformatic tools [4]. A complementary review in *Microorganisms* by González and colleagues focused on the promise of long-read methods for microbiome analysis in general [3]. The present review differs from both in its focus on the specific question of what is recovered in practice when methods are compared on matched samples, and in its explicit engagement with the biases that occur after recent chemistry and bioinformatics improvements. Table 1 summarises the benchmarking studies, highlighting the evidence that supports each argument discussed in the subsequent sections.

## 3. Technical Background: What Each Method Does and What It Systematically Misses

### 3.1. Short-Read 16S rRNA Amplicon Sequencing

Short-read 16S sequencing amplifies one or two hypervariable regions of the 16S rRNA gene and sequences them on Illumina platforms that produce paired end reads of 2 × 250 or 2 × 300 bp. V3–V4 and V4 are the most used regions in soil work. The approach has extremely low per-base error rates and a mature denoising and taxonomic classification ecosystem anchored by tools such as DADA2 and QIIME 2 [17,18].

Its systematic limitations are well recognised. First, reads are too short to span the full 16S gene, so taxonomic assignment is usually limited to the genus level, with species-level assignments being unreliable [8]. Second, primer choice biases which taxa are amplified. Different hypervariable regions capture different fractions of bacterial diversity [19,20]. Third, MiSeq, regardless of bioinformatics method, tends to have a higher approximation of Actinobacteria and Bacteroidetes, and both high- and low-GC contents can have a negative bias in the MiSeq platform [6]. Fourth, even within the V4 region there is a well-documented subset of bacteria, notably some SAR11 lineages, which require primer revisions to detect at all [15].

### 3.2. Full-Length 16S rRNA Gene Sequencing with ONT

Full-length 16S sequencing on ONT MinION, GridION, or PromethION platforms targets the entire 1500 bp 16S gene using primer pairs such as 27F/1492R. The 1500 bp product covers all nine variable regions of the gene and in principle allows classification up to the species level. The main concern with ONT has been per-read accuracy. Early R9 flow cells had reported accuracies in the range of 85–95%, which placed ONT 16S data at a significant disadvantage to Illumina for taxonomic classification [9].

The R10.4.1 flow cell, combined with Q20+ kit chemistry and updated basecallers, was designed specifically to close this gap. With the R10.4.1 flow cell and Q20+ kit chemistry, ONT now approaches per-read accuracies that close the historical gap with Illumina; we evaluate this claim in detail in Section 5 [10]. Independent evaluations generally confirm this. In a three-platform soil benchmark, ONT produced results that closely matched those of PacBio, suggesting that ONT’s inherent sequencing errors do not significantly affect the interpretation of well-represented taxa [5]. The central remaining issues for ONT full-length 16S in soil is not raw accuracy but rather persistent platform-specific biases, which we examine in detail in Section 5.

### 3.3. Shotgun Metagenomic Sequencing

Shotgun metagenomic sequencing sequences all accessible DNA in a sample rather than a targeted gene. It enables functional profiling, recovery of MAGs, strain-level resolution, and detection of non-bacterial community members (archaea, viruses, eukaryotes) that 16S amplification miss. It is also considerably more expensive per sample than amplicon sequencing and requires orders of magnitude more reads per sample of soil because of the high community diversity [16].

Long-read and hybrid shotgun approaches have transformed what is possible in this space. Recent soil studies combining Nanopore long-reads with Illumina short-reads have recovered hundreds of high- and medium-quality MAGs from single soil samples, with long reads particularly valuable for contig contiguity and the resolution of repetitive regions [13,14]. Whether such MAG recovery captures a representative portion of soil diversity, or methodically misses high-diversity populations, is an active question [12].

## 4. Direct Head-to-Head Evidence: What Different Methods Recover from the Same Soil

The most decisive evidence on method choice come from studies that apply multiple methods to the same DNA extracts. Two recent studies are central here: a three-platform benchmark by Veselovsky and colleagues using Illumina, PacBio, and ONT R10.4.1 on three soil types [5], and a two-platform benchmark by Stevens and colleagues using Illumina MiSeq and ONT MinION on two agricultural soils and a mock community [6]. Together, they establish the pattern of agreement and disagreement that defines the current state of the field.

### 4.1. What the Methods Agree on

Dominant taxa and between-sample structure are robust to platform choice. In the Veselovsky et al. three-platform benchmark, the top six bacterial genera identified across ONT and PacBio platforms were *Brevitalea*, *Solirubrobacter*, *Baekduia*, *Vicinamibacter*, *Bacillus,* and *Gaiella*, and these dominant genera remained consistent across all sequencing depths [5]. Over half of all identified species were detected by both long-read platforms at every sequencing depth tested. The Procrustes test, which is a statistical procedure that quantifies the similarity between two ordination configurations by minimising the sum-of-squares distance after rotation and scaling, returned a *p*-value of 0.001 at every sequencing depth, indicating that the community structure was actually similar between platforms even when individual species counts differed in the samples.

Between-site and between-method differences are also largely robust to platform choice. In the Stevens et al. two-platform benchmark applied to agricultural soils from Colorado and Oregon, at all taxonomic levels, the full-length MinION method had the highest similarity to the short MiSeq method with DADA2 correction, with 73.2%, 69.3%, 74.1%, 79.3%, 79.4%, and 82.3% of taxa at the phyla, class, order, family, genus and species levels respectively, showing similar patterns in differences between the sites [6]. In practical terms, if a researcher’s question is “do these two soils differ, and if so, in what taxa,” the answer is likely to be similar regardless of whether short-read or full-length 16S is used, though not identical.

### 4.2. What the Methods Disagree on

Alpha diversity estimates differ systematically across platforms, and the differences are large enough to matter. Illumina reads accounted for the identification of 232 genera, while ONT detected 545, with 188 genera shared between both platforms in the Veselovsky et al. study [5]. Whether this represents genuine improved sensitivity by ONT or inflated richness from sequencing errors is one of the main methodological questions in the field. The Stevens et al. study is particularly informative here because it directly tested this question with a mock community of known composition. The MiSeq platform with DADA2 error correction resulted in the correct estimate of mock community species richness and much lower alpha diversity for soils, while MinION full-length sequencing without equivalent error correction produced inflated richness estimates [6]. When filtering was applied to remove taxa below a 0.07% relative abundance threshold, MinION and MiSeq richness estimates converged. This strongly suggests that a substantial share of ONT-detected rare taxa in soils are sequencing or classification artefacts rather than genuine low-abundance organisms.

Relative abundances of individual phyla differ analytically and reproducibly between platforms. The Stevens et al. study showed that the MiSeq platform was enriched for Actinobacteria, Chloroflexi, and Gemmatimonadetes, while the MinION platform was enriched for Acidobacteria, Bacteroidetes, Firmicutes, Proteobacteria, and Verrucomicrobia regardless of the bioinformatics pipeline used [6]. Similar platform-specific biases in respiratory samples have since been documented, where ONT over-represented certain taxa such as *Enterococcus* and *Klebsiella* while under-representing others such as *Prevotella* and *Bacteroides* [7]. In a study of the human nasal microbiota, a particularly striking case was recognised where ONT severely underestimated *Corynebacterium* abundance due to primer mismatches with several *Corynebacterium* species [8]. The soil microbiome contains many high-GC organisms (Actinobacteria, for example), and the platform biases observed in benchmark studies have practical consequences; a researcher reporting “Actinobacteria-dominated soil” based on Illumina V3–V4 data may be describing an amplification bias as much as a biological fact.

The choice of hypervariable region is at least as important as the choice of platform. This is worth emphasising because it is often looked at as a minor methodological footnote. In the Veselovsky et al. study, microbial community analysis ensured clear clustering of samples based on soil type regardless of sequencing technology, with the only exception being the V4 region, where no soil-type clustering was observed (*p* = 0.79) [5]. A V4-only Illumina study of these three soils would have concluded that they did not differ in microbial community composition. A V3–V4 study of the same samples, or any long-read study, would have concluded that they differed significantly.

### 4.3. Evidence from Non-Soil Benchmarks

Benchmarks in non-soil systems provide useful additional context but must be interpreted with care, because soil-specific features (such as high diversity, high humic acid content, low microbial biomass per unit DNA after extraction) amplify some biases and suppress others. In a rabbit gut comparison, Biada and colleagues applied Illumina MiSeq V3–V4, PacBio HiFi full-length and ONT MinION full-length to the same samples and noted different levels of taxonomic resolution across platforms [21]. In respiratory samples from both human and pig models, Macip and colleagues found that Illumina captured greater species richness, while community evenness remained comparable between platforms, and beta diversity differences were significant in pig samples but not in human samples, suggesting that sequencing platform effects are more noticeable in complex microbiomes [7]. The last observation is particularly relevant for soil because soil is the most complex microbiome of all: the platform-effect magnitude observed in soil should, if anything, exceed that observed in these systems.

## 5. The R10.4.1 Question: How Much Has the Chemistry Update Changed?

A large portion of the historical case against ONT for microbiome work can be seen through the per-read error rates that were genuinely problematic on R9 flow cells. The R10.4.1 flow cell, which was introduced commercially in 2022, combined with updated basecaller models (Guppy, then Dorado with HAC and SUP modes), significantly narrowed the accuracy gap with Illumina. Understanding the extent of this change is important for any analytical method choice decision.

### 5.1. What R10.4.1 Has Improved

Zhang and colleagues provided the conclusive initial characterisation of R10.4.1 for 16S amplification and sequencing work. Their study compared NovaSeq, PacBio Sequel II, and ONT PromethION across both R9.4.1 and R10.4.1 flow cells on mock communities and three microbiome types, where they have reported that the error rate in ONT R10.4.1 is significantly lower, especially when deletions are involved, getting to more than 99% accuracy [10]. In the Veselovsky et al. soil benchmark using R10.4.1 with the Dorado basecaller, both HAC and SUP basecalling modes produced identical precision, recall, and F1 scores on the ZymoBIOMICS standard (0.91, 0.77, and 0.83 respectively), though the SUP mode required considerably more computational time and resources [5].

### 5.2. What R10.4.1 Has Not Improved

Two observations qualify the straightforward “R10.4.1 solves the accuracy problem” reading. First, even with R10.4.1 chemistry, ONT in soil still filters out substantially more reads than PacBio does. The percentage of reads filtered out was lower for PacBio (0.52%) compared to ONT (16.7%), reflecting the higher sequencing error rate associated with ONT [5]. This is not a large enough difference to change biological inferences in most cases, but it does mean that an ONT run needs to produce more raw reads than an equivalent PacBio run to reach the same post-filtering depth.

Second, higher per-read accuracy does not address platform biases that originate upstream of basecalling. Primer-specific amplification biases, library preparation biases, and taxonomic classifier biases are there regardless of how precisely the basecaller converts the signal to sequence. The Heikema et al. observation that ONT primers fail to amplify certain *Corynebacterium* species is not an accuracy problem and will not be solved by flow cell chemistry improvements [8]. Likewise, the GC-content biases that favour certain phyla on Illumina’s sequencing-by-synthesis chemistry are not primer-specific and are not improved by ONT chemistry updates. The practical implication is that the R10.4.1 update has made ONT a competitive option for full-length 16S in soil, but it has not made ONT and Illumina interchangeable. Platform biases remain; they are just different biases.

### 5.3. The Role of Bioinformatics: Emu and Downstream Tooling

The application of algorithms developed specifically for microbial community profiling from full-length 16S rRNA gene sequences, such as Emu, ensures the generation of fewer false positives and negatives than other methods, thus reducing the error rates [10]. Emu, which uses an expectation maximisation algorithm for read-by-read error correction against a reference database, has become the standard for ONT full-length 16S classification in the benchmarking literature [5,6,7]. Researchers considering R10.4.1 for soil work should not compare chemistry-era papers directly because the entire downstream pipeline of data analysis has changed in parallel.

Lastly, a recent methodological study validated a flexible barcoded nanopore workflow using the v14 version kits, comparing performance against MALDI-TOF identification of bacterial isolates and against Illumina shotgun sequencing for community profiling, and found that the workflow produced accurate identifications for both applications [22]. While this research was not conducted on soil, it supports the broader picture that current-generation ONT workflows are sufficient for the community profiling of complex samples.

## 6. Shotgun Metagenomics: What Is Actually Assembled from Soil

The shotgun metagenomic picture for soil is different from the amplicon picture because the limiting step is rarely sequencing accuracy and is almost always sequencing depth combined with the inherent difficulty of assembling a community with thousands of species, some with near identical genomic segments.

Hybrid assembly approaches that combine short-read accuracy with long-read contiguity have generated the most comprehensive soil MAG recoveries to date. Bağcı and colleagues applied approximately 148 billion base pairs of Nanopore long-read data, together with 122 billion base pairs of Illumina short-read data, to a single forest soil sample and reconstructed 837 MAGs, including 466 meeting high- and medium-quality standards [14]. Belliardo and colleagues demonstrated that short-read-derived coverage information significantly improves the binning of HiFi long-read assemblies from tunnel-cultivated soil [13]. The common thing is that, for soil, neither short-read nor long-read shotgun alone recovers the available information. Short-reads lack the contiguity to resolve repetitive and mobile genetic elements in complex communities. Long-reads, even at high accuracy, benefit substantially from short-read coverage information for binning into MAGs. Hybrid approaches are expensive and bioinformatically challenging, but they currently define the state of the art for soil metagenomics.

A recent evaluation by Berg and colleagues compared short-read and long-read metagenome assemblies in a natural soil community and found systematic bias in the recovery of high-diversity populations [12]. This is an important result to any enthusiast that says long-read sequencing is usually a better option than that of short-read one. Both have methodical blind spots, and the blind spots are not the same. High-diversity populations (those containing many closely related strains at low individual abundance) are particularly difficult for both approaches and are recovered with systematic biases that differ by method.

This leads to two key takeaways. First, “shotgun metagenomics” is not a single method and different shotgun approaches recover different organisms in soil. A short-read shotgun study and a long-read shotgun study of the same soil will not produce the same MAGs, and the differences are not random. Second, published shotgun soil microbiome surveys that use only one approach should be interpreted as describing the organisms that the approach can recover, not the full community. Meta-analyses that pool MAGs across studies using different sequencing strategies are particularly susceptible to this kind of bias.

## 7. An Evidence-Based Decision Framework

The evidence reviewed here supports a framework for method choice that is tied to the specific research question, rather than to a generic ranking of platforms. We outline five common question types and methods, supported by current evidence for each.

For broad community composition examinations where the question is “what taxa are present and how do they differ between samples,” short-read 16S on Illumina with DADA2 error correction and the V3–V4 region is currently the most defensible choice. It is the best-benchmarked, cheapest, and most interpretable against the existing literature. V4 alone should be avoided in soil work given its low ability to discriminate soil types [5]. Primer choice matters and should be justified, not defaulted to.

For up to genera-level resolution questions, full-length 16S on ONT R10.4.1 with comparison against recent reference database is supported by current benchmarking evidence. The Veselovsky et al. soil benchmark demonstrated that this approach produces results comparable to PacBio on dominant taxa, at a fraction of the per-sample cost [5]. Researchers should be aware that alpha diversity estimates will be inflated relative to Illumina-with-DADA2, unless filtering is applied, and should report both unfiltered and filtered diversity estimates.

For functional profiling, MAG recovery, and detection of non-bacterial community species, shotgun metagenomics is required, and for soil samples specifically, hybrid long-plus-short-read sequencing currently produces the most complete recovery of community members. Cost and bioinformatic complexity mean this is not practicable for all studies, but for research with conclusions dependent on functional or strain-level information, 16S alone is insufficient regardless of read length.

For time-sensitive or field-based applications, ONT has unique advantages. The portability of the MinION platform, the ability to basecall in real time, and the cheap sequencer options make ONT a great choice in many field contexts [9,22]. Soil monitoring, rapid pathogen detection in agricultural locations, and remote-location work are natural applications.

For low-biomass, atypical, or non-agricultural soil settings, the picture is less clear and benchmarking is currently inadequate. This includes urban soils, industrially contaminated soils, aquatic-margin soils, soils from specially protected areas and nature reserves, permafrost samples, and paired rhizosphere-versus-bulk soil comparisons. Existing benchmarks have been conducted almost exclusively on agricultural and forest soils, and the platform biases observed in those settings cannot be assumed to simplify for soils with very different physicochemical properties or microbial biomass. We return to this as a major gap in Section 8.

A note on hybrid 16S-plus-shotgun designs: many of the studies reviewed here used both amplicon and shotgun sequencing on the same samples to get a fuller picture than either provides alone [14]. This is an expensive but increasingly common study design, and should be considered in studies where both community composition and function are important.

## 8. Gaps in Current Benchmarking

Despite the growing frame of comparison studies, several gaps limit confident method choice in soil work and point to priorities for future methodological research.

First, almost all three-platform soil benchmarks had been conducted on agricultural or forest soils. Other land-use categories of major scientific and applied importance remain effectively un-benchmarked, including urban and residential soils, industrial-zone soils with elevated contaminant loads, water-resource and aquatic-margin soils, soils from specially protected areas and nature reserves, and permafrost samples. Rhizosphere-versus-bulk paired comparisons across the same sequencing methods also remain limited. Given that platform biases are amplified in more complex microbiomes [7], we cannot assume that patterns observed in agricultural soil generalise to all soil types and land uses. Systematic benchmarking across these categories is, in our view, the most important methodological priority for the soil microbiome field.

Second, research looking at the R10.4.1 chemistry is still in its early stages. The Zhang et al. study [10] and the Veselovsky et al. study [5] are two of the few published direct evaluations on environmental samples, and they are both single-laboratory studies. Inter-laboratory reproducibility of R10.4.1 results on matched soil samples has not been properly evaluated.

Third, benchmarking of soil ONT shotgun metagenomics specifically, as opposed to 16S amplicon or hybrid approaches, remains limited. The Berg et al. systematic bias observation [12] is vital but, is, to our knowledge, not yet replicated across independent soil datasets.

Fourth, no published benchmark has, to our knowledge, methodically assessed the same soil samples across DNA extraction methods, sequencing platforms, and bioinformatic pipelines in an entirely crossed design. Soil DNA extraction is known to introduce substantial bias independently of sequencing [23,24]. The relative magnitude of extraction, sequencing, and pipeline biases in soil has not been quantified in a single study, which means current practice is largely based on the implied assumption that platform choice matters more than extraction choice. This assumption is reasonable but not recognised.

Fifth, soil-specific reference databases and taxonomic classifiers remain underdeveloped. Much of the current classification relies on databases built primarily from human-associated microbiomes, which means that well-represented soil taxa may be poorly resolved, and true soil taxa may be misclassified as database-adjacent organisms. This is a problem that cuts across all sequencing methods, but is particularly critical for ONT full-length 16S, where getting to almost species-level assignment is the headline benefit.

## 9. Conclusions

The question “which sequencing method is best for soil microbiomes?” does not have a single answer, and treating it as if it does has produced the literature in which studies of similar questions reach different conclusions because of procedural choices that are often unstated or under-justified. Head-to-head benchmarking on matched soil samples establishes a clearer frame: dominant taxa and between-sample structure are generally conserved across methods, while alpha diversity estimates, rare taxon detection, relative phylum abundances, and the ability to distinguish closely related samples are all substantially shaped by method choice.

The R10.4.1 chemistry has genuinely reduced the per-read accuracy gap between ONT and Illumina and has made full-length 16S a practical choice for soil laboratories that could not previously access up-to-species-level resolution. It has not, however, made platform biases disappear. Researchers using R10.4.1 should expect inflated alpha diversity estimates relative to DADA2-corrected Illumina data, differences in phylum-level relative abundances compared to Illumina, and some persistent primer-related gaps that the chemistry update does not address.

Long-read shotgun metagenomics, particularly in hybrid configurations with short-reads, has transformed what is recoverable from soil at the MAG level, but has its own systematic biases. Short-read and long-read shotgun approaches recover different portions of a soil community, and neither alone captures the full picture in high-diversity environments.

The practical recommendation for soil microbiome researchers is to select methods on the basis of the research question, to use filtering and quality-control approaches appropriate to the method used, and to treat cross-study meta-analyses that pool data across methods with corresponding caution. As the sequencing technology landscape continues to evolve, periodic re-evaluation of these recommendations will be essential.

## Figures and Tables

**Table 1:** Summary of head-to-head benchmarking studies reviewed.

Study	Sample Type	Technologies Compared	Mock Included?	Key Finding Relevant to This Review
Veselovsky et al., 2025 [5] Front Microbiol	Three soil types (agricultural)	Illumina (V3–V4, V4) + PacBio (full + trimmed) + ONT R10.4.1 (full)	Yes (ZymoBIOMICS)	ONT R10.4.1 comparable to PacBio on dominant taxa; V4 Illumina alone fails to cluster samples by soil type (*p* = 0.79). Central three-platform soil benchmark.
Stevens et al., 2023 [6] Sci Rep	Two agricultural soils (Colorado, Oregon)	Illumina MiSeq (V3–V4) + ONT MinION (V3–V4 and full 16S)	Yes	Phylum-level platform biases regardless of bioinformatics pipeline; MinION full + MiSeq + DADA2 agree on 70–82% of taxa across levels. Second central soil benchmark.
Berg et al., 2025 [12] NAR Genom Bioinform	Natural soil community	Short-read shotgun + long-read shotgun	No (natural community)	Systematic bias in recovery of high-diversity populations; short-read and long-read shotgun recover different MAGs from the same soil.
Bağcı et al., 2025 [14] GigaScience	Single forest soil sample	ONT long-read (148 Gb) + Illumina short-read (122 Gb), hybrid assembly	No	Ultra-deep hybrid sequencing recovered 837 MAGs (466 high/medium quality); even at this depth, rarefaction analysis indicates soil community remains under-sampled.
Belliardo et al., 2025 [13] bioRxiv (preprint)	Tunnel-cultivated soil	PacBio HiFi long-read + Illumina short-read	No	Short-read-derived coverage significantly improves binning of HiFi long-read assemblies; justifies hybrid approach for soil MAG recovery.
Zhang et al., 2023 [10] Appl Environ Microbiol	Three microbiome types (incl. soil)	NovaSeq + PacBio Sequel II + ONT R9.4.1 + ONT R10.4.1 (all full-length 16S)	Yes	R10.4.1 achieves ~99% accuracy; significantly lower error rate than R9.4.1, especially for deletions. Foundational R10.4.1 characterisation.
Heikema et al., 2020 [8] Genes (MDPI)	Human nasal microbiota + isolates	Illumina (V5–V6) + ONT R9.2/R9.4 (full 16S)	Yes (isolates)	Genus-level agreement between platforms, but ONT primers fail to amplify some *Corynebacterium* species—primer-mismatch bias that chemistry updates do not fix.
Macip et al., 2025 [7] Sci Rep	Respiratory (human VAP + pig model)	Illumina NextSeq (V3–V4) + ONT R10.4.1 (full 16S, Kit V14)	No	Illumina higher richness; ONT over-represents *Enterococcus*, *Klebsiella*, under-represents *Prevotella*, *Bacteroides*; platform effects larger in complex (pig) samples than simple (human).
Biada et al., 2025 [15] Front Microbiomes	Rabbit gut (soft faeces)	Illumina MiSeq (V3–V4) + PacBio HiFi (full) + ONT MinION (full)	No	Three-way methodological comparison; different levels of taxonomic resolution across platforms. Informative but rabbit gut, not soil.
Dommann et al., 2024 [16] mSystems	Bacterial isolates + human stool	ONT v14 chemistry kits vs. MALDI-TOF (isolates) and Illumina shotgun (stool)	No	Validates ONT v14 workflow for both isolate identification and community profiling; supports current-generation ONT as fit-for-purpose.
Chavan et al., 2022 [9] Heliyon	Soil + Ag/Ti/Zn nanoparticles	ONT MinION (full-length 16S), single-platform applied study	No	Example application of ONT full 16S to environmental soil research (contaminant impact).
Fujiyoshi et al., 2020 [11] Sci Rep	Mock communities + environmental samples	ONT MinION (full 16S) PCR condition optimisation, Illumina MiSeq comparison	Yes (3 mocks)	Establishes optimised PCR conditions for full-length nanopore 16S; foundational method paper for ONT amplicon work.

Notes. Rows 1–5 are the most directly relevant soil-specific benchmarks. Rows 6–12 are supporting methodological studies in non-soil or single-platform contexts cited throughout the review. Mock community inclusion is flagged because it is the single most decisive control for distinguishing true biological differences from platform artefacts. Technologies column lists the sequencing platforms used.

## Data Availability

No new data were created or analyzed in this study. Data sharing is not applicable to this article.
